# TMAO accelerates cellular aging by disrupting endoplasmic reticulum integrity and mitochondrial unfolded protein response

**DOI:** 10.1007/s00018-024-05546-z

**Published:** 2025-01-21

**Authors:** Fahimeh Varzideh, Emanuele Farroni, Urna Kaunsakar, Mahaba Eiwaz, Stanislovas S. Jankauskas, Gaetano Santulli

**Affiliations:** 1 Department of Medicine, Wilf Family Cardiovascular Research Institute, Institute for Neuroimmunology and Inflammation (INI), Einstein Institute for Aging Research, New York, NY USA; 2 Department of Molecular Pharmacology, Fleischer Institute for Diabetes and Metabolism (FIDAM), Einstein-Mount Sinai Diabetes Research Center (ES-DRC), Albert Einstein College of Medicine, Albert Einstein College of Medicine, 1300 Morris PARK AVENUE, New York, NY 10461 USA

Metabolic disorders are functionally linked to skeletal fragility and early mortality in older adults [1]. For instance, obesity suppresses bone growth, over-stimulates glucocorticoid activity and accelerates bone degradation; diabetes mellitus triggers inflammation and disrupts bone balance [1]. Recent observations suggest that imbalances within the microbiome can cause gut barrier deterioration, eventually leading to bone loss [2]. Trimethylamine N-oxide (TMAO), a metabolite produced by gut microbes (Fig. 1), raises oxidative stress and inflammation in the bone, further increasing the risk of osteoporosis in obese individuals [2]. Additionally, endoplasmic reticulum (ER) stress disrupts protein folding, initiating an unfolded protein response (UPR) that contributes to osteoporosis [3]. However, the exact role of TMAO in osteoblast activity and osteoporosis onset was hitherto quite unclear. In this sense, filling a long-standing knowledge gap in the field, in the current issue of *CMLS*, Yu-Han Lin and collaborators [4] demonstrate the catabolic effects of TMAO on bone maintenance during osteoporosis caused by obesity or estrogen deficiency. They elegantly elucidate the molecular basis of the inhibitory effects of TMAO on osteoblasts, showing that it disrupts ER integrity and mitochondrial UPR^mt^, thereby accelerating cell aging and reducing the mineralized extracellular matrix [4].

**Fig. 1:**
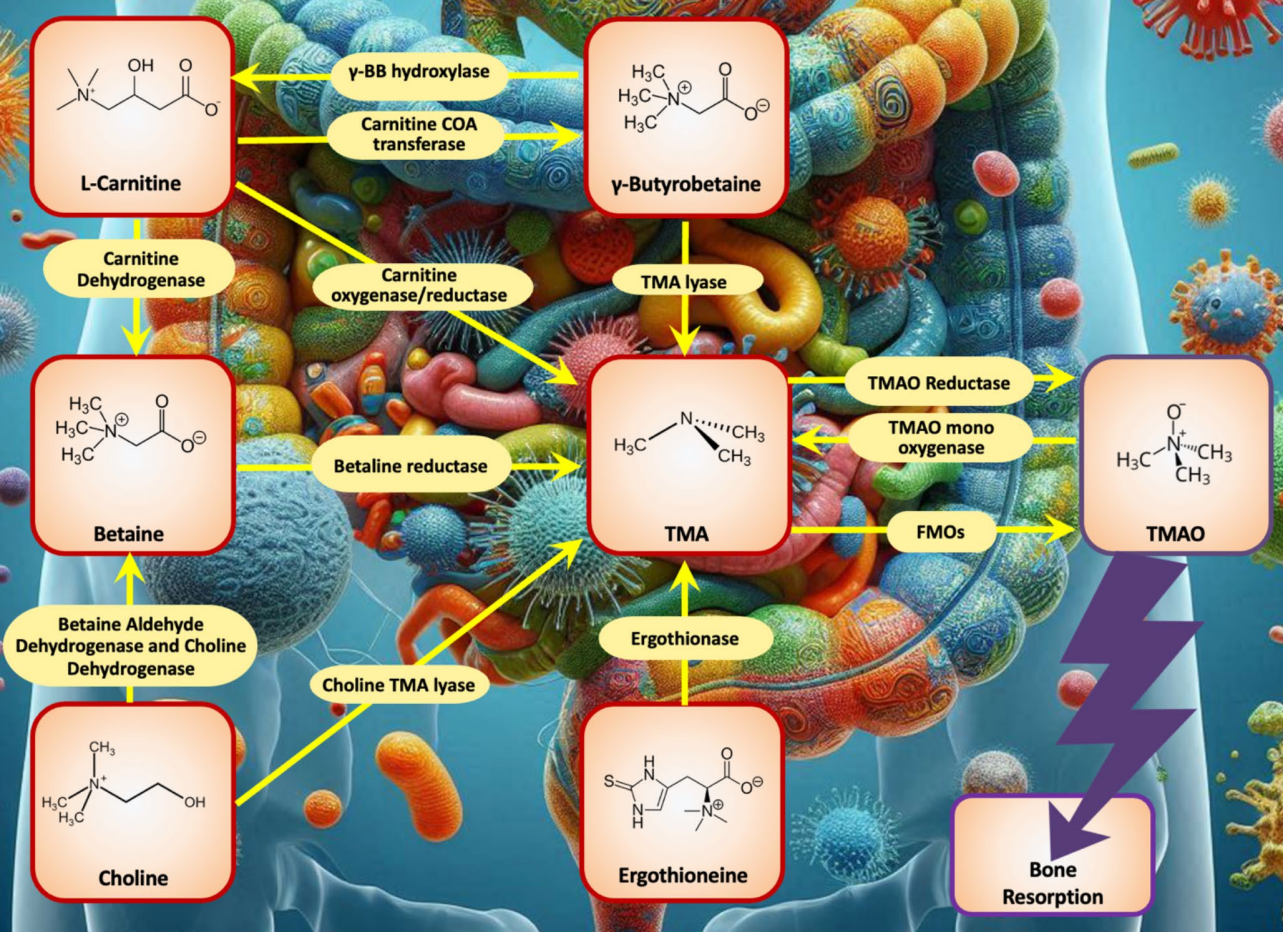
Main molecular pathways leading to the biosynthesis of TMAO by the gut microbiome γ-BB: γ-Butyrobetaine; FMAOs: Flavin monooxygenases; TMA: trimethylamine; TMAO: Trimethylamine N-oxide

Their findings align with other studies indicating that gut microecological alterations can affect immune response and brain-gut-bone interactions, fostering osteoporotic changes [5, 6]. In fact, mounting evidence suggests that gut microbes release extracellular vesicles and/or metabolites that can influence osteogenic differentiation [7, 8]. Gut barrier deterioration (including diminished mucin, reduced tight junction proteins, and elevated IL-17) has been associated with bone degradation in obesity [9, 10]. Intriguingly, Yu-Han Lin and colleagues specifically correlated beneficial gut bacteria like *Lactobacillus* and *Akkermansia* with improved bone traits [4], including mineral density, trabecular integrity, and balanced bone turnover. Consistent with these findings, probiotic *Lactobacillus* intake supports antioxidant capabilities [11], curbing bone degradation, whereas reduced *Akkermansia* levels have been shown to accelerate bone loss [12].

Metabolomic analyses of serum profiles revealed that a disrupted L-carnitine metabolism, linked to gut microbiota dysbiosis, markedly contributes to bone deterioration [4]. L-carnitine supports mitochondrial fatty acid metabolism, necessary for osteogenesis [13]. Considering that low serum levels of L-carnitine represent a prevailing feature in patients with osteoporosis suggests its potential in mitigating bone loss. In agreement with these observations, L-carnitine is metabolized into trimethylamine (TMA) by gut microorganisms and flavin containing monooxygenase 3 (FMO3) is known to oxidize TMA into TMAO [14], as shown in Fig. 1. These metabolomic findings underscore the complexity of the gut-bone connection.

Hence, TMAO seems to act as a functional gut-derived metabolite substantially contributing to osteoporotic changes in conditions of obesity and estrogen deficiency; in particular, TMAO promotes bone loss by tipping the balance toward osteoclast-mediated resorption. Little is known about the precise molecular effects of TMAO on bone turnover; of interest, TMAO has been suggested to shift bone marrow mesenchymal stem cells toward fat rather than bone-forming cells [15]. TMAO activates PERK, disrupting ER stability and autophagy processes, eventually leading to osteoblast aging. Reducing PERK-mediated stress in osteoblasts was found to support cell survival under TMAO exposure, further highlighting its suppressive role in bone formation [4].

It is important to emphasize that the effects of TMAO on the synthesis of mineralized matrix components are context-dependent. In cardiovascular tissues, TMAO promotes osteogenic activity by enhancing Runx2 transcription, leading to matrix calcification in vascular smooth muscle cells via NLRP3 inflammasome activation [16].

TMAO also triggers mitochondrial stress, which has been implied in regulating osteogenesis of aortic valve cells [17]. TMAO has been shown to impede a number of mitochondrial activities, including energy production, respiration, and oxidative phosphorylation [18]. Moreover, this microbial metabolite may disrupt the mitochondrial UPR (UPR^mt^) by triggering misfolding of its key regulator ATF5 and can suppress mineralized matrix synthesis in models of osteoporosis [4]. Strikingly, rescuing UPR^mt^ via nicotinamide ribose restores mitochondrial energy levels [4], enabling osteoblasts to produce mineralized matrix despite TMAO exposure, confirming that TMAO inhibits bone anabolism in osteoporosis. Consistent with these observations, UPR^mt^ has been shown to support mitochondrial function and bone stem cell differentiation in response to metabolic stress [19, 20].

Despite its novelty and potential translational relevance for clinicians, the work is not exempt from limitations. For instance, the authors did not rule out that other gut-derived metabolites could also impact osteoblast activity and bone homeostasis. Furthermore, TMAO might influence additional mitochondrial metabolic pathways, including the Krebs cycle, glycolysis, and/or fatty acid biosynthesis.

In conclusion, TMAO may hinder osteoblast function by inducing ER stress and misfolding of ATF5 in UPR^mt^; thus, gut dysbiosis and metabolic imbalances can promote bone loss. Further studies on gut microbiota transplantation may provide insights on its bone-protective effects, potentially slowing osteoporosis progression. Dedicated investigations are also warranted to determine whether these pathways are also present in other clinical conditions that have been previously linked to TMAO, including diabetes, atherosclerosis, thrombosis, heart failure, and metabolic syndrome [21–25].

## Data Availability

Enquiries about data availability should be directed to the authors.
